# Supplementary material for: Genome-wide analyses of self-reported empathy: correlations with autism, schizophrenia, and anorexia nervosa
Source: Transl Psychiatry. 2018 Mar 12;8:35. doi: 10.1038/s41398-017-0082-6 (PMC5845860; doi:10.1038/s41398-017-0082-6)
Supplement: Supplementary file 1 — Supplementary Information [file 41398_2017_82_MOESM1_ESM.docx]

Supplementary Information

Genome-wide analyses of self-reported empathy: correlations with autism, schizophrenia, and anorexia nervosa

Table of Contents

[Supplementary Figure 1: Manhattan Plot (A) and QQ Plot (B) of the non-stratified GWAS analysis 2](#_Toc497128448)

[Supplementary Figure 2: Manhattan Plot (A) and QQ Plot (B) of the females-only GWAS analysis 3](#_Toc497128449)

[Supplementary Figure 3: Manhattan Plot (A) and QQ Plot (B) of the males-only GWAS analysis 4](#_Toc497128450)

[Supplementary Figure 4: Regional Association plots 5](#_Toc497128451)

[Supplementary Figure 5: Regional association plots for Chr11p12 10](#_Toc497128452)

[Details about the PGC_iPSYCH autism GWAS meta-analyses 11](#_Toc497128453)

## Supplementary Figure 1: Manhattan Plot (A) and QQ Plot (B) of the non-stratified GWAS analysis


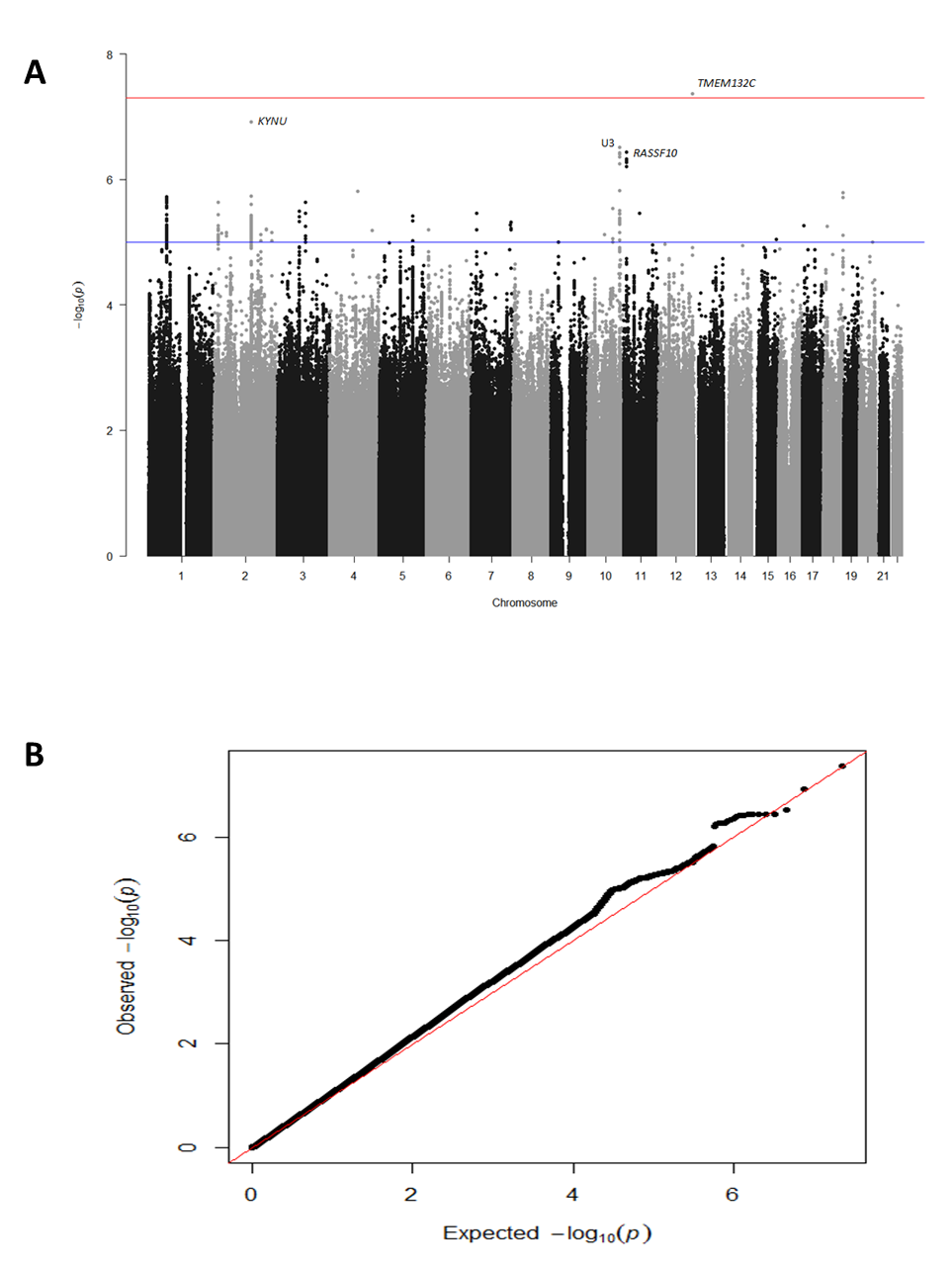


*λ_GC_ = 1.092, LDSR intercept = 1.0007±0.0066*

## Supplementary Figure 2: Manhattan Plot (A) and QQ Plot (B) of the females-only GWAS analysis


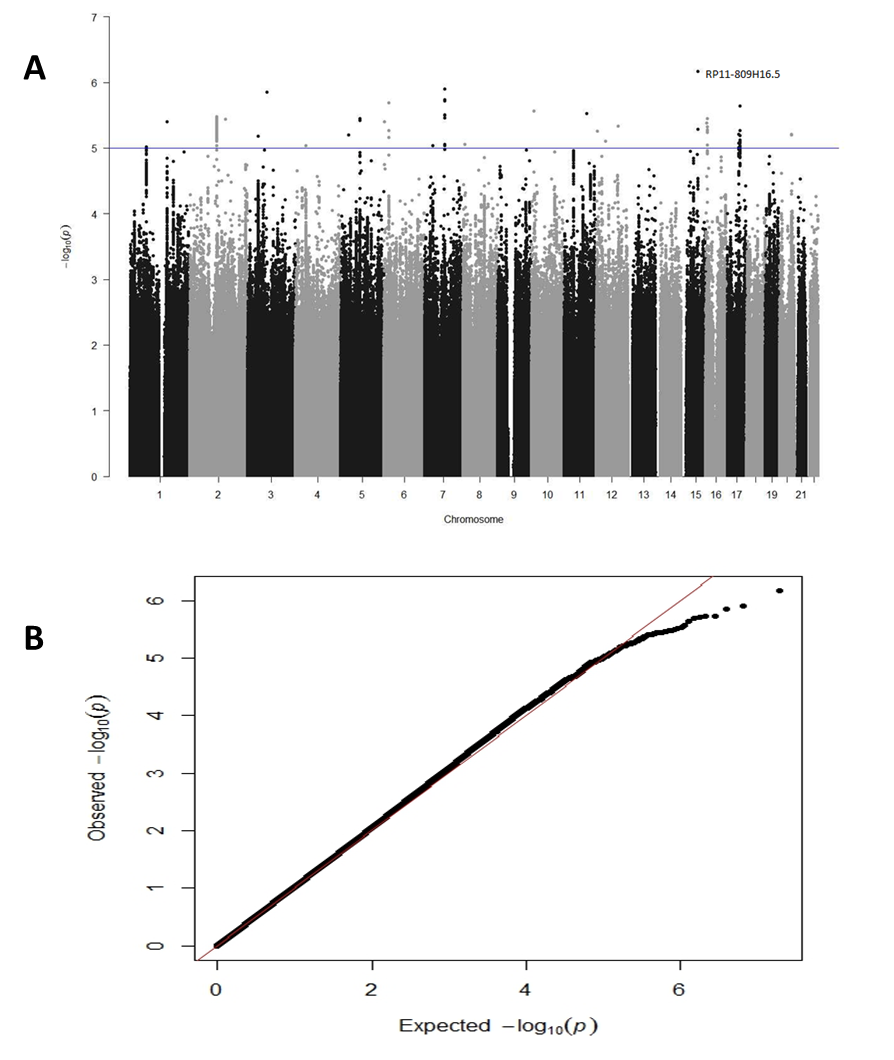


*λ_GC_ = 1.049, LDSR intercept = 1.0015±0.0064*

## Supplementary Figure 3: Manhattan Plot (A) and QQ Plot (B) of the males-only GWAS analysis


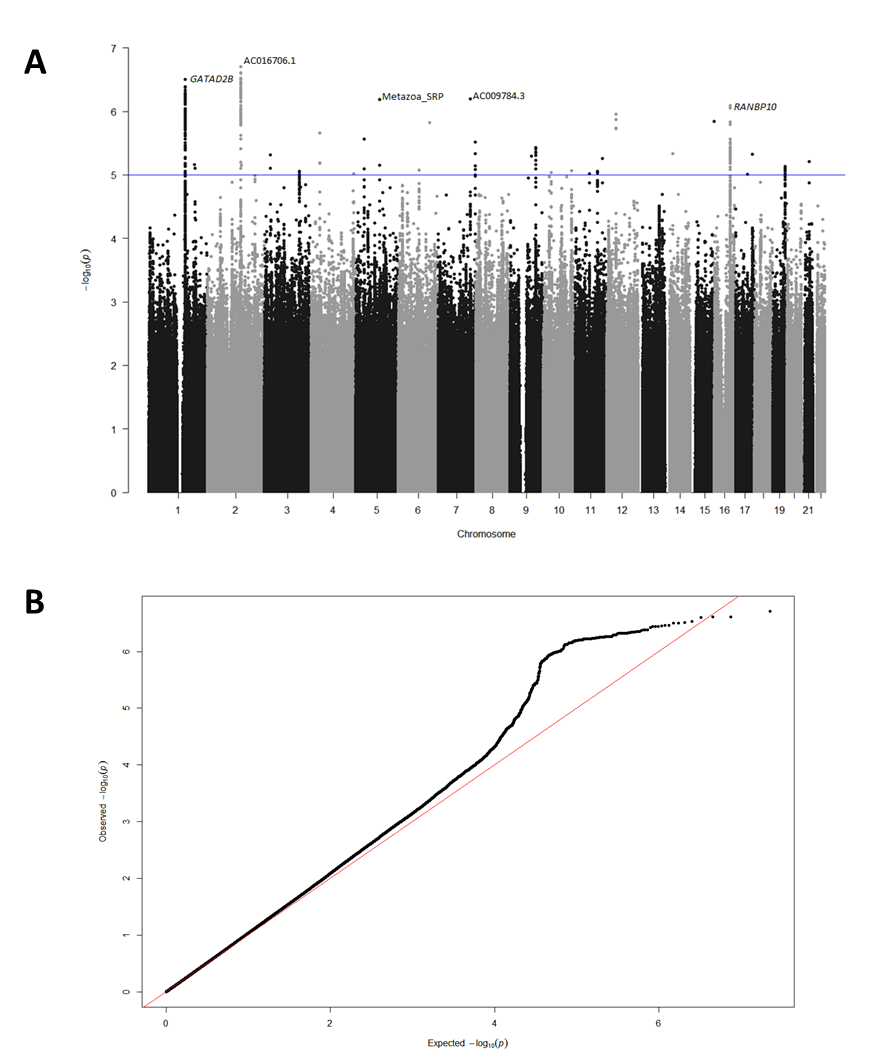


*λ_GC_ = 1.051, LDSR intercept = 0.99±0.0055*

## Supplementary Figure 4: Regional Association plots

(LD data was unavailable for rs201219357 and rs201219357, and so these SNPs were not plotted).

A. Regional association plot for rs4882760 (Non-stratified GWAS)


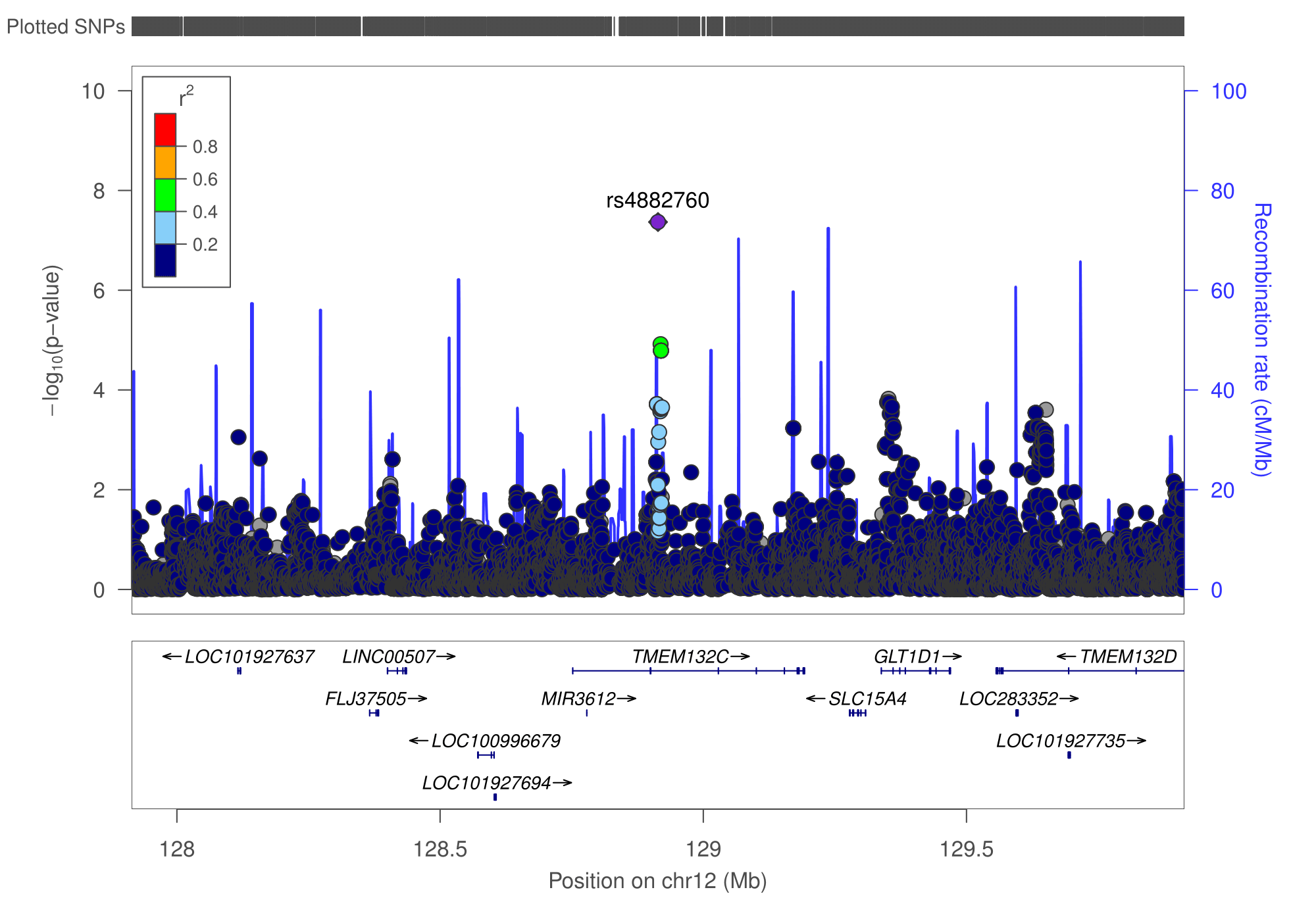


B. Regional association plot for rs1141090 (Non-stratified GWAS)
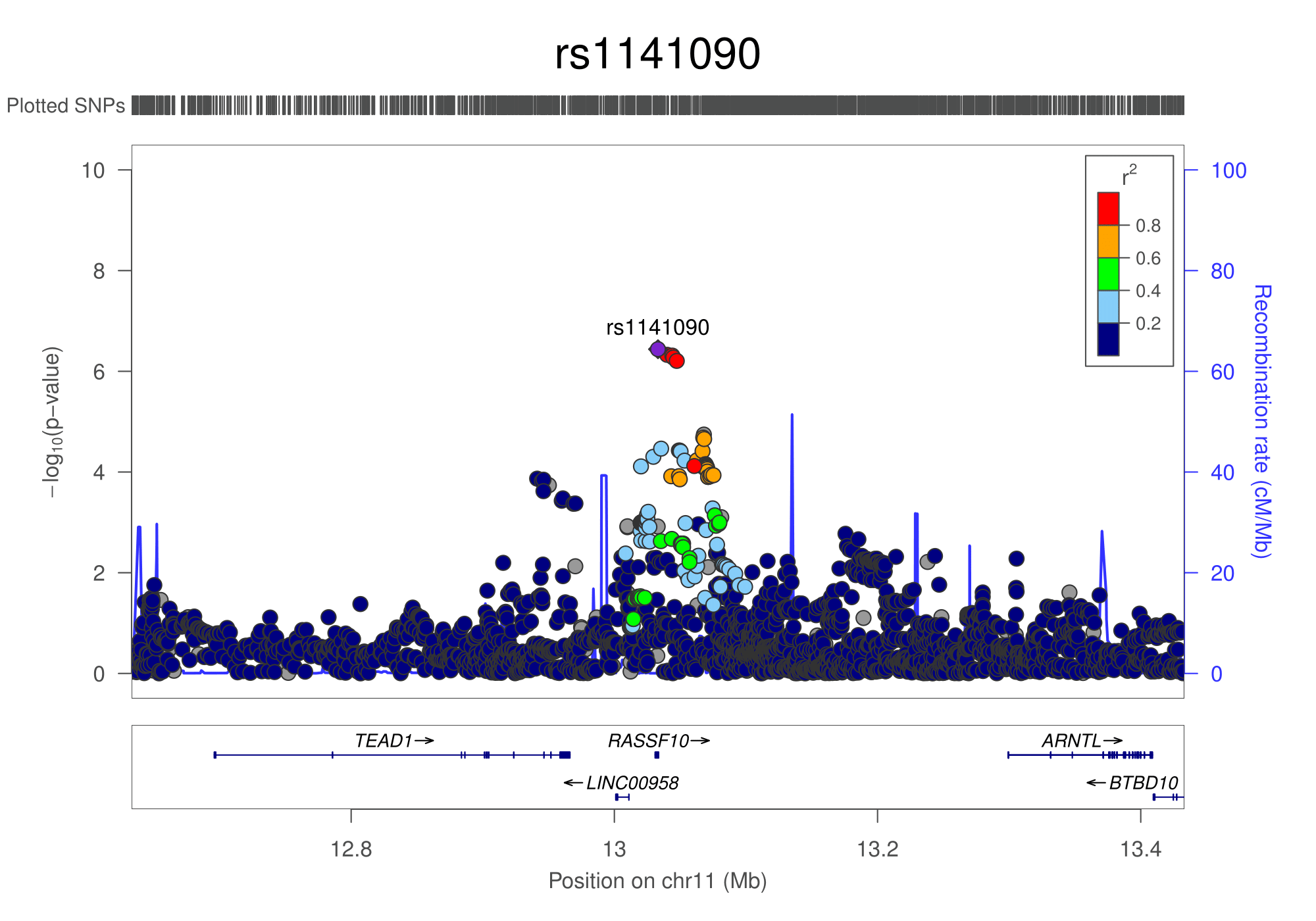


C. Regional association plot for rs2089401 (Males-only GWAS)


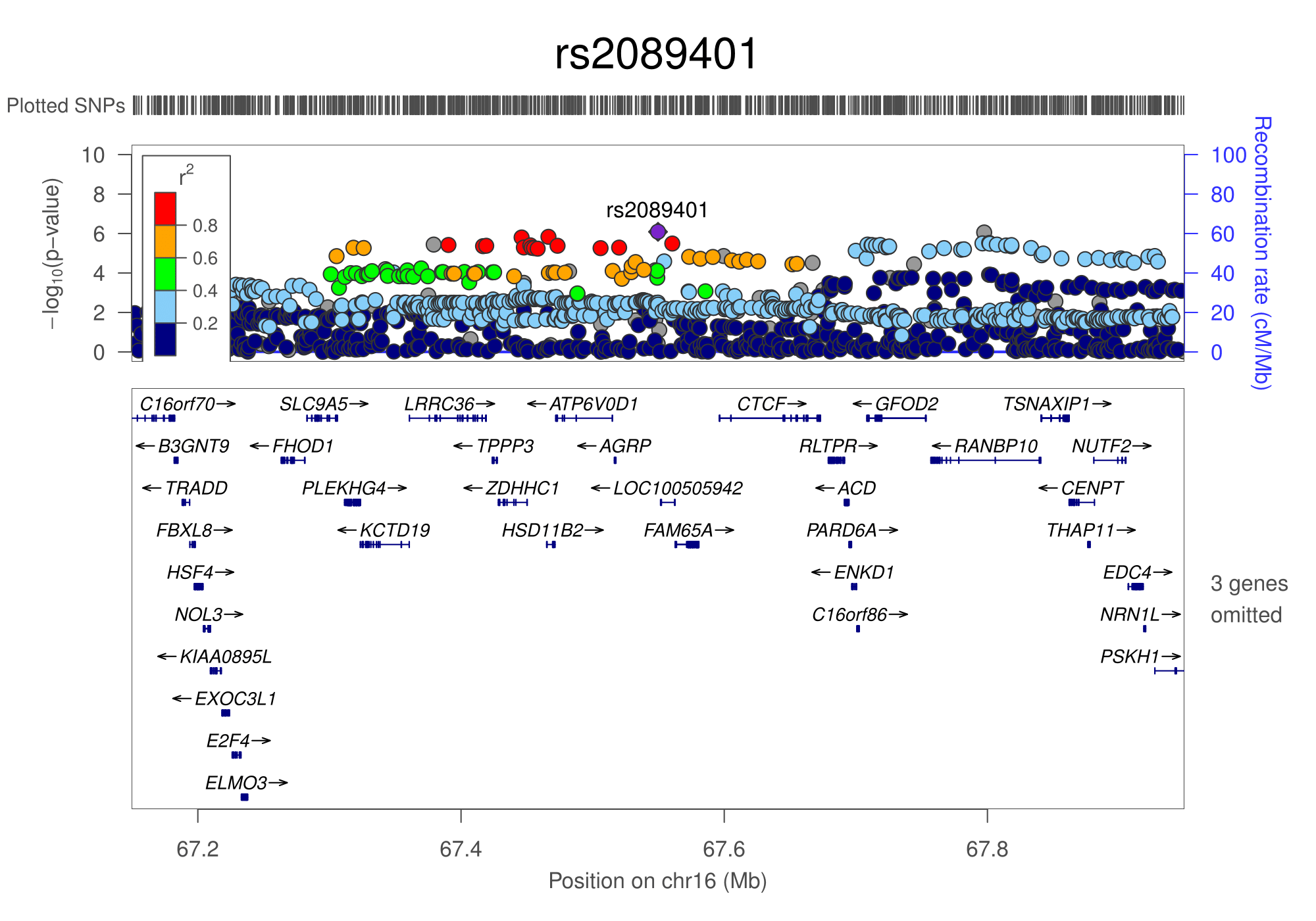


D. Regional association plot for rs10265275 (Males-only GWAS)


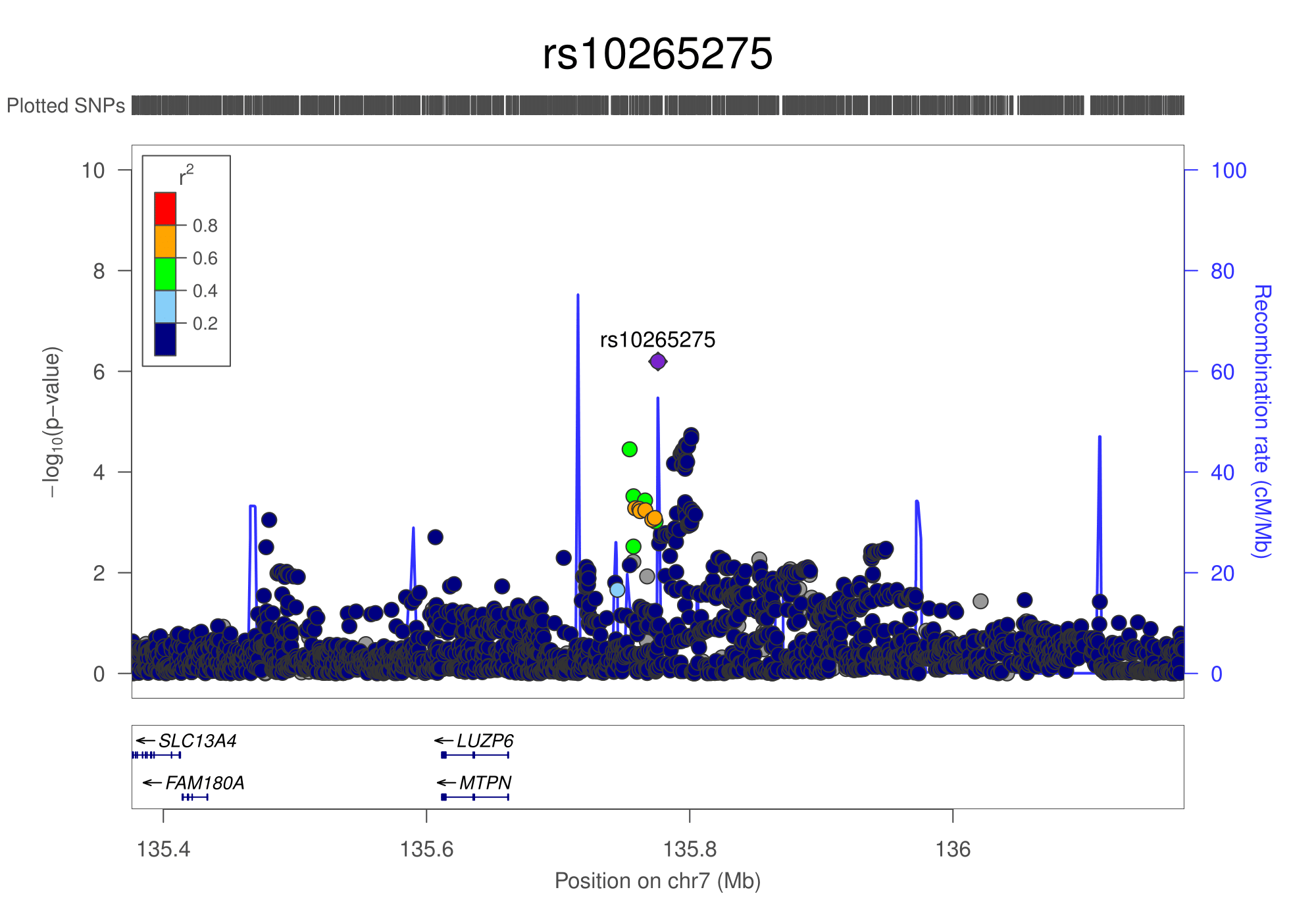


E. Regional association plot for rs11264567 (Males-only GWAS)


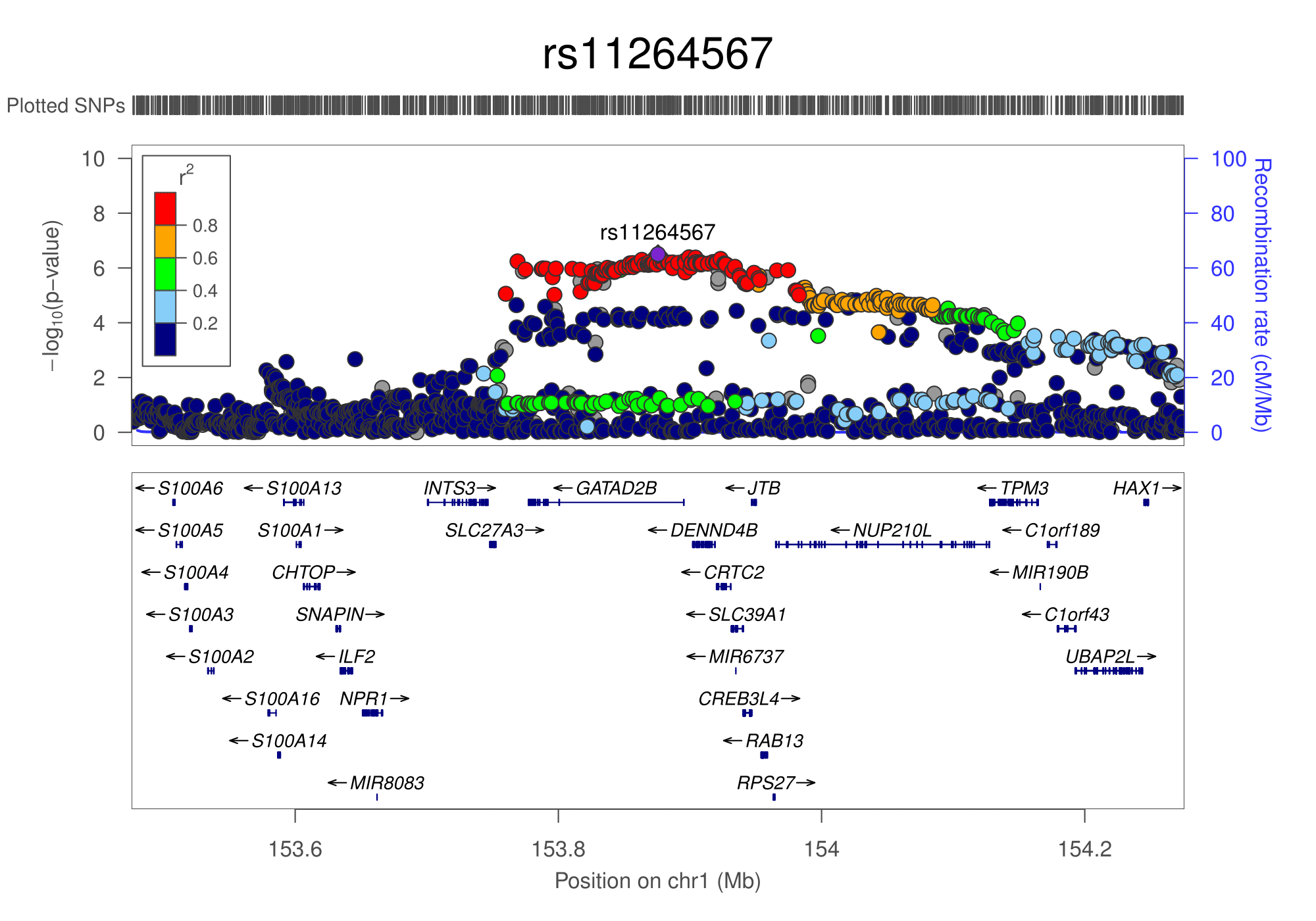


F. Regional association plot for rs75171949 (Males-only GWAS)


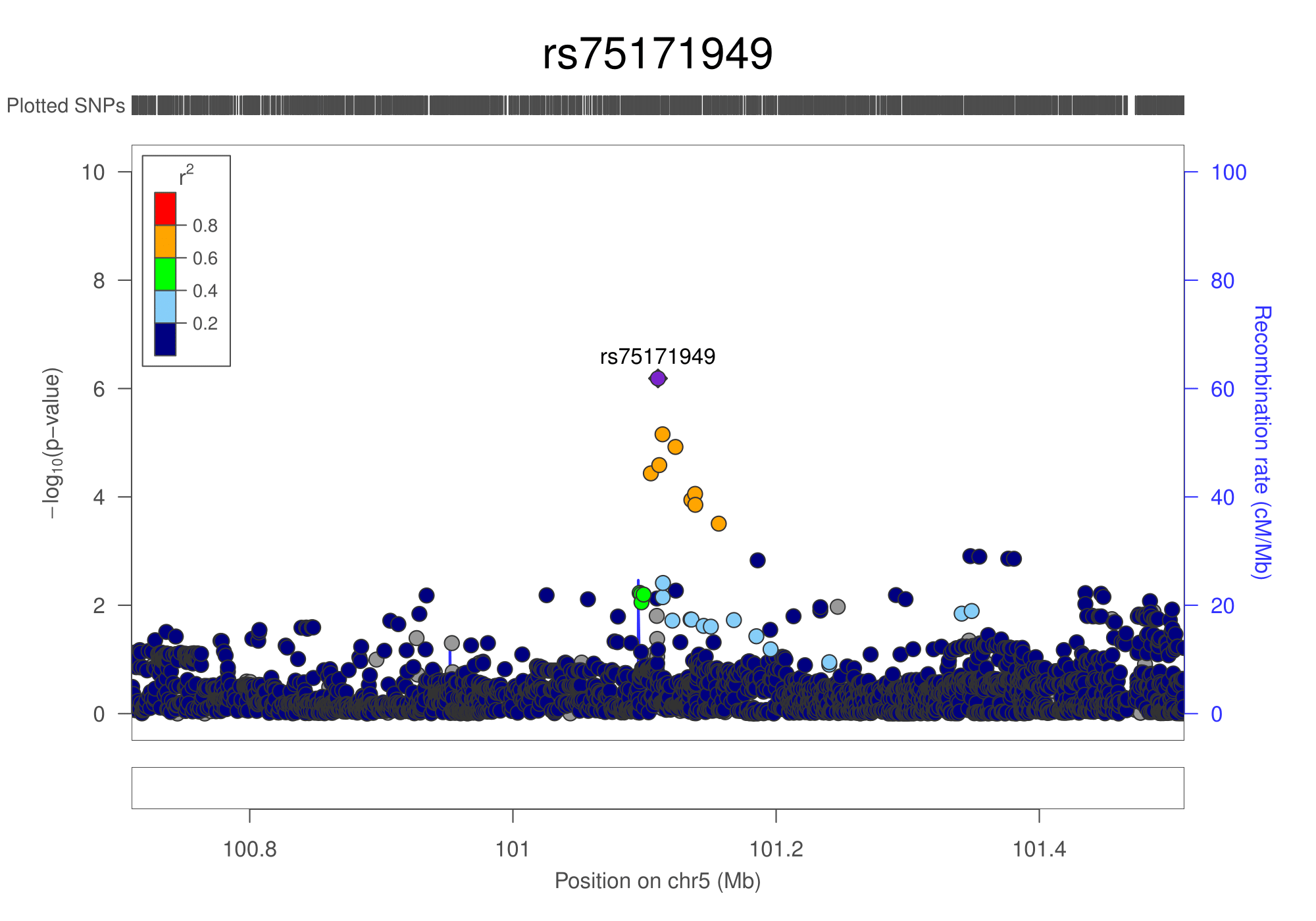


G. Regional association plot for rs76891664 (Males-only GWAS)


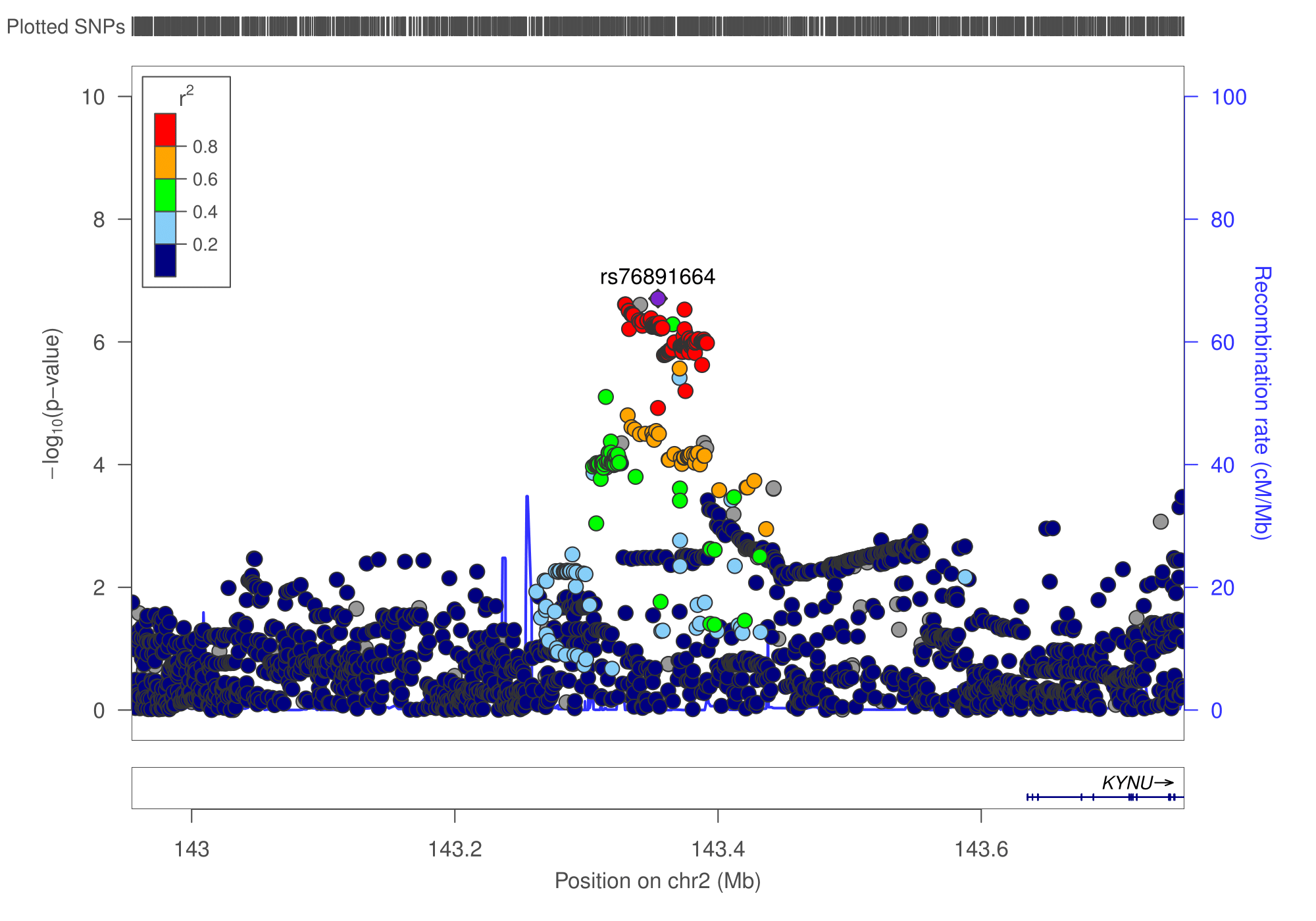


H. Regional association plot for rs146838217 (Non-stratified GWAS)


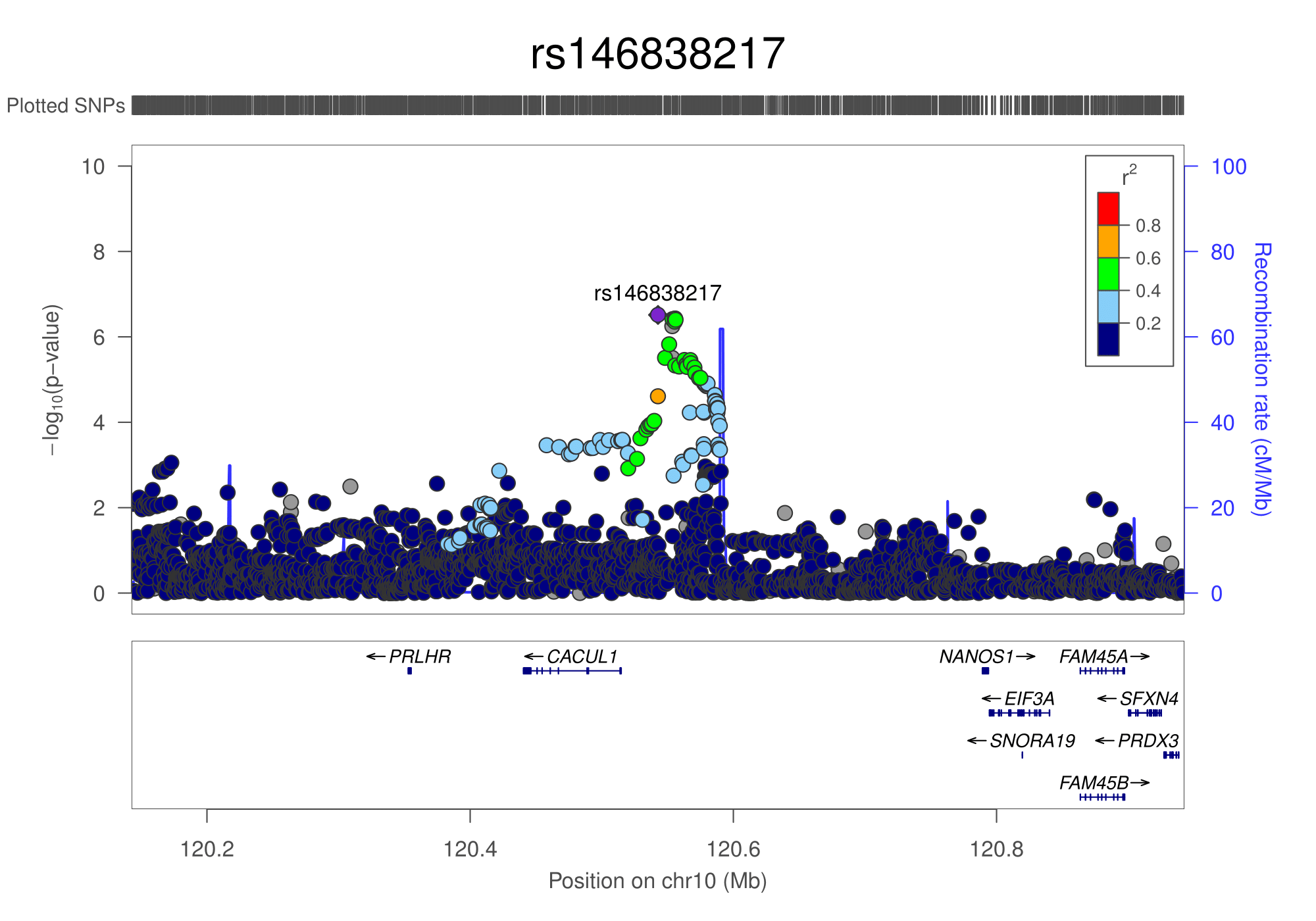


I. Regional association plot for rs189163756 (Non-stratified GWAS)


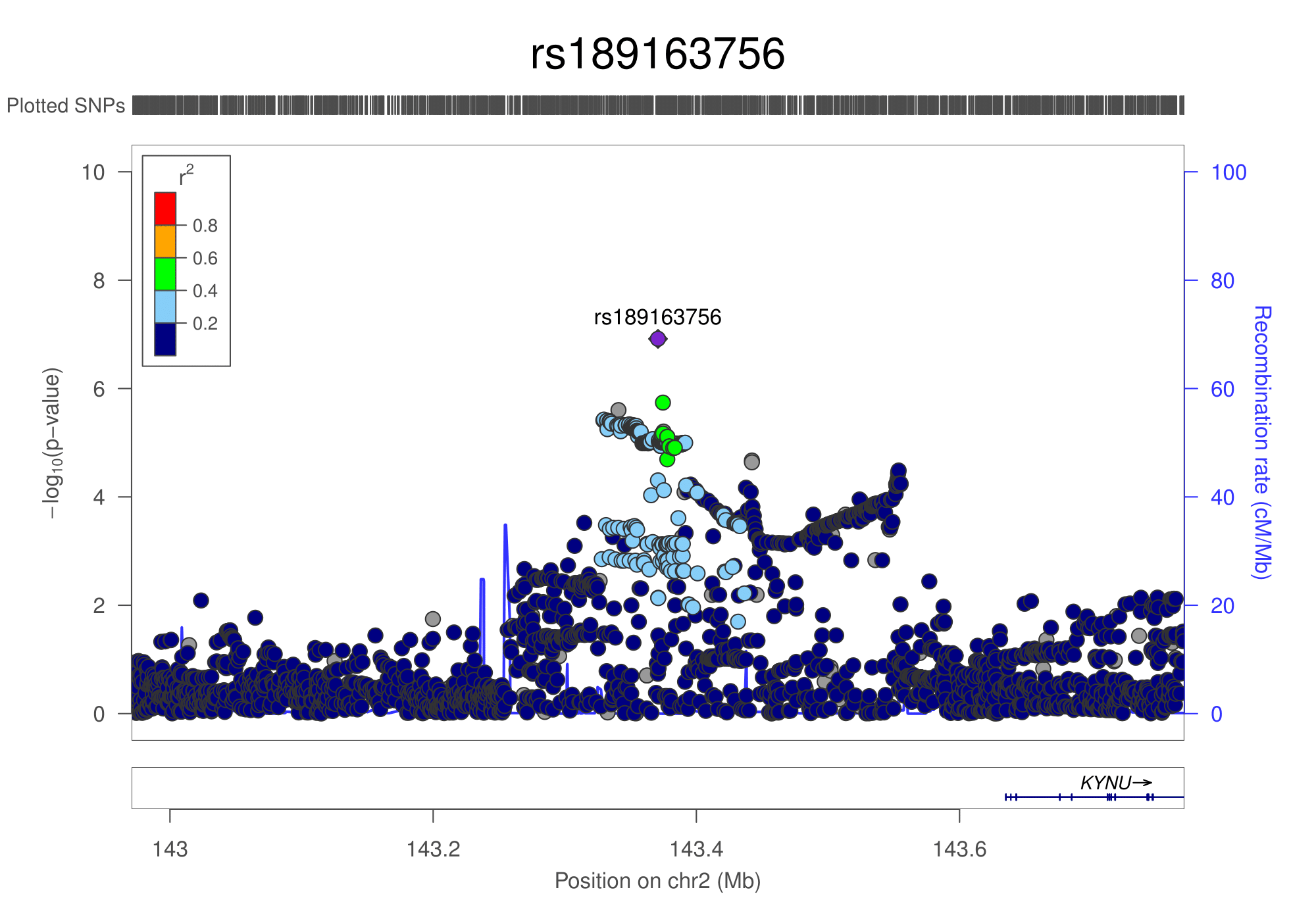


## Supplementary Figure 5: Regional association plots for Chr11p12

A. Regional association plot for Chr11: 39,000,000- 41,000,000 (EQ, non-stratified).


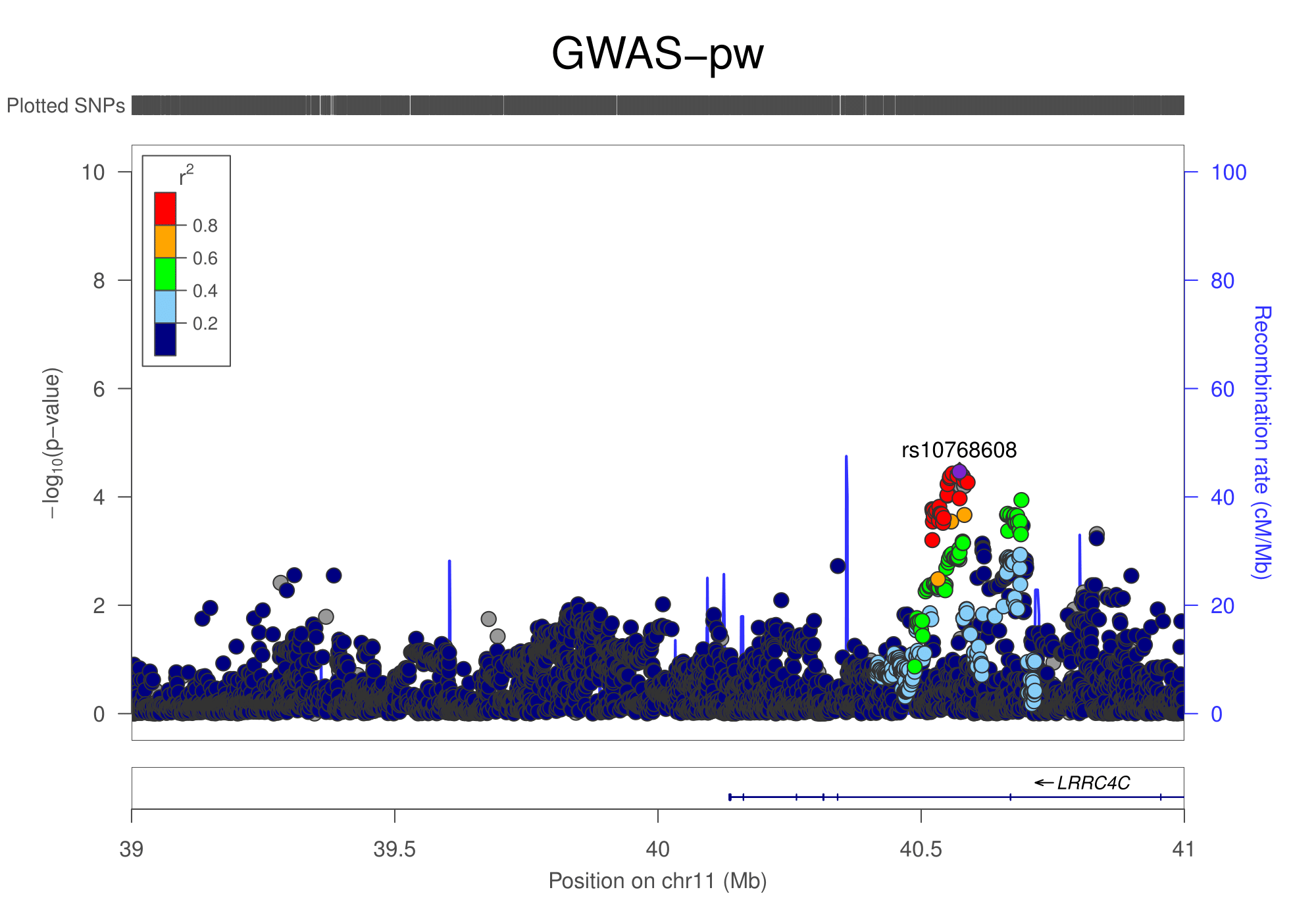


B. Regional association plot for Chr11: 39,000,000- 41,000,000 (Anorexia, non-stratified).
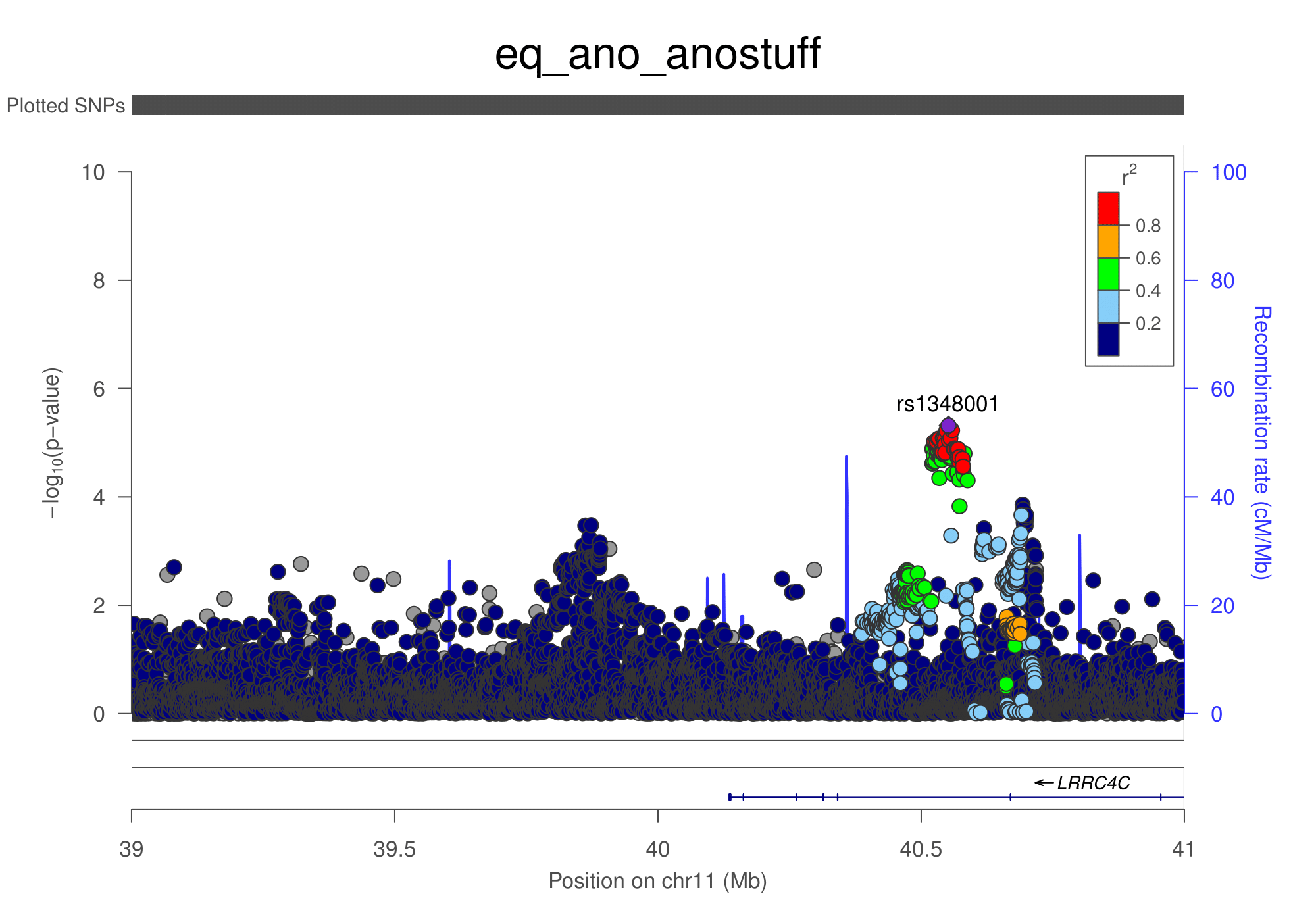


## Details about the PGC_iPSYCH autism GWAS meta-analyses

*Samples:* The ASD summary statistics are based on a meta analysis of the results based on 5305 trios of European ancestry from the PGC autism sample and 13076 cases and 22664 controls of European ancestry from the iPSYCH autism GWAS which is part of the iPSYCH case-cohort sample^1^. Briefly, the iPSYCH ASD sample is extracted from the birth cohorts consisting of all children born in Denmark between May 1st 1981 and December 31st 2005. Eligible were singletons born to a known mother and resident in Denmark on their one-year birthday. Cases were defined from the Danish Psychiatric Central Research Register as those having an ASD diagnosis (ICD codes F84.0, F84.1, F84.5, F84.8 or F84.9) given no later than 2013. The controls constitute a random sample from the complement. The samples were linked using the unique personal identification number to the Danish Newborn Screening Biobank. Genotypes are available on 14970 cases and 26125 controls.

A detailed description of the PGC sample is available on the PGC web site: <https://www.med.unc.edu/pgc/files/resultfiles/PGCASDEuro_Mar2015.readme.pdf> and even more details are provided elsewhere^2^. Briefly, five cohorts provided genotypes (n denote the number of trios for which genotypes were available): The Geschwind Autism Center of Excellence (ACE; n = 391), the Autism Genome Project (AGP; n = 2272), the Autism Genetic Resource Exchange (AGRE; n = 974), the NIMH Repository (<https://www.nimhgenetics.org/available_data/autism/>), the Montreal/Boston Collection (MONBOS; n = 1396, and the Simons Simplex Collection (SSC; n = 2231). The trios were analyzed as cases and pseudo controls.

*Methods:* Data processing and QC was conducted according to the standards employed by the PGC Statistical Analysis Group and carried out using their pipeline Ricopili^3^. The data was processed separately in the 23 genotyping batches in the case of iPSYCH and for each cohort in the PGC sample. Phasing was achieved using SHAPEIT^4^ and imputation done by IMPUTE2^5^ with haplotypes from the 1000 Genomes Project, phase 3 (1kGP3) as reference. Trio samples were imputed as a case-pseudo-controls design.

Prior to principal component analysis (PCA) regions of high LD were excluded and genotypes were pruned down to a set of roughly 30k markers by pruning in a sliding window fashion using plink 1.9. Using plink’s identity by state analysis, pairs of subjects were identified with $\hat{\pi}>0.2$ and one subject of each such pair excluded at random keeping cases when possible. PCA was carried out using smartPCA^6^. In iPSYCH a subsample of European ancestry was selected using a ellipsoid in the space of the first 3 principal components (PCs) centred and scaled using the mean and 8 standard deviation of the PCs restricted to the subsample whose parents and grandparents were all known to have been born in Denmark (n=31500). In the PGC sample a Euclidian distance measure weighted by the variance explain for each of the first 3 PCs. Individuals more distant than 10 standard deviations from the combined CEU and TSI HapMap reference populations were excluded. For both iPSYCH and PGC samples we conducted a secondary PCA to provide covariates for the association analyses.

Association analyses were done applying plink 1.9 to the imputed dosage data for each iPSYCH batch at a time and each PGC subcohort at a time. The results were subsequently meta-analysed using METAL^7^ (July 2010 version) employing an inverse variance weighted fixed effect model. Prior to release we filtered the summary statistics allowing only markers with an imputation info score $\geq0.7$, maf $\geq0.01$ and an effective sample size of at least 70% of the maximum. The effective sample size was estimated from the number of cases, Nca, and controls, Nco, contributing to the individual regression as 2*Nca*Nco/(Nca+Nco).

*References*

1 Pedersen CB, Bybjerg-Grauholm J, Pedersen MG, Grove J, Agerbo E, Bækved-Hansen M *et al.* The iPSYCH2012 case–cohort sample: new directions for unravelling genetic and environmental architectures of severe mental disorders. *Mol Psychiatry* 2017. doi:10.1038/mp.2017.196.

2 The Autism Spectrum Disorders Working Group of The Psychiatric Genomics Consortium. Meta-analysis of GWAS of over 16,000 individuals with autism spectrum disorder highlights a novel locus at 10q24.32 and a significant overlap with schizophrenia. *Mol Autism* 2017; **8**: 21.

3 Ripke S, Neale BM, Corvin AP, Walters JTR, Farh K-H, Holmans PA *et al.* Biological insights from 108 schizophrenia-associated genetic loci. *Nature* 2014; **511**: 421–7.

4 Delaneau O, Marchini J, Zagury J-F. A linear complexity phasing method for thousands of genomes. *Nat Methods* 2011; **9**: 179–181.

5 Howie BN, Fuchsberger C, Stephens M, Marchini J, Abecasis GR. Fast and accurate genotype imputation in genome-wide association studies through pre-phasing. *Nat Genet* 2012; **44**: 955–9.

6 Price AL, Patterson NJ, Plenge RM, Weinblatt ME, Shadick NA, Reich D. Principal components analysis corrects for stratification in genome-wide association studies. *Nat Genet* 2006; **38**: 904–909.

7 Willer CJ, Li Y, Abecasis GR. METAL: fast and efficient meta-analysis of genomewide association scans. *Bioinformatics* 2010; **26**: 2190–1.

## List of authors and their affiliations for the iPSYCH-Broad autism group

| **Group/sample source** | **EMAIL** | **Surname** | **First name** | **Middle name/Initials** | **Professional degrees** | **Affiliations** |
| --- | --- | --- | --- | --- | --- | --- |
| iPSYCH | [ea@econ.au.dk](mailto:ea@econ.au.dk) | Agerbo | Esben |  | DMSci | The Lundbeck Foundation Initiative for Integrative Psychiatric Research, iPSYCH, Denmark ; National Centre for Register-Based Research, Aarhus University, Aarhus, Denmark ; Centre for Integrated Register-based Research, Aarhus University, Aarhus, Denmark |
| iPSYCH | [tda@biomed.au.dk](mailto:tda@biomed.au.dk) | Als | Thomas | Damm | PhD | The Lundbeck Foundation Initiative for Integrative Psychiatric Research, iPSYCH, Denmark ; Centre for Integrative Sequencing, iSEQ, Aarhus University, Aarhus, Denmark ; Department of Biomedicine - Human Genetics, Aarhus University, Aarhus, Denmark |
| Broad/MHG | [rbell@broadinstitute.org](mailto:rbell@broadinstitute.org) | Belliveau | Rich |  |  | Stanley Center for Psychiatric Research, Broad Institute of Harvard and MIT, Cambridge, Massachusetts, USA |
| iPSYCH | [JOGR@ssi.dk](mailto:JOGR@ssi.dk) | Bybjerg-Grauholm | Jonas |  |  | The Lundbeck Foundation Initiative for Integrative Psychiatric Research, iPSYCH, Denmark ; Center for Neonatal Screening, Department for Congenital Disorders, Statens Serum Institut, Copenhagen, Denmark |
| iPSYCH | [MABH@ssi.dk](mailto:MABH@ssi.dk) | Bækved-Hansen | Marie |  |  | The Lundbeck Foundation Initiative for Integrative Psychiatric Research, iPSYCH, Denmark ; Center for Neonatal Screening, Department for Congenital Disorders, Statens Serum Institut, Copenhagen, Denmark |
| iPSYCH | [anders@biomed.au.dk](mailto:anders@biomed.au.dk) | Børglum | Anders | D. | MD, PhD | The Lundbeck Foundation Initiative for Integrative Psychiatric Research, iPSYCH, Denmark ; Centre for Integrative Sequencing, iSEQ, Aarhus University, Aarhus, Denmark ; Department of Biomedicine - Human Genetics, Aarhus University, Aarhus, Denmark |
| Broad/MHG | [fcerrato@broadinstitute.org](mailto:fcerrato@broadinstitute.org) | Cerrato | Felecia |  |  | Stanley Center for Psychiatric Research, Broad Institute of Harvard and MIT, Cambridge, Massachusetts, USA |
| Broad/MHG | [chambert@broadinstitute.org](mailto:chambert@broadinstitute.org) | Chambert | Kimberly |  |  | Stanley Center for Psychiatric Research, Broad Institute of Harvard and MIT, Cambridge, Massachusetts, USA |
| Broad/MHG | [cchurch@broadinstitute.org](mailto:cchurch@broadinstitute.org) | Churchhouse | Claire |  | PhD | Analytic and Translational Genetics Unit, Department of Medicine, Massachusetts General Hospital and Harvard Medical School, Boston, USA ; Stanley Center for Psychiatric Research, Broad Institute of Harvard and MIT, Cambridge, Massachusetts, USA ; Program in Medical and Population Genetics, Broad Institute of Harvard and MIT, Cambridge, Massachusetts, USA |
| Broad/MGH | [mjdaly@atgu.mgh.harvard.edu](mailto:mjdaly@atgu.mgh.harvard.edu) | Daly | Mark | J. | PhD | Analytic and Translational Genetics Unit, Department of Medicine, Massachusetts General Hospital and Harvard Medical School, Boston, USA ; Stanley Center for Psychiatric Research, Broad Institute of Harvard and MIT, Cambridge, Massachusetts, USA ; Program in Medical and Population Genetics, Broad Institute of Harvard and MIT, Cambridge, Massachusetts, USA |
| iPSYCH | [ditte@biomed.au.dk](mailto:ditte@biomed.au.dk) | Demontis | Ditte |  | PhD | The Lundbeck Foundation Initiative for Integrative Psychiatric Research, iPSYCH, Denmark ; Centre for Integrative Sequencing, iSEQ, Aarhus University, Aarhus, Denmark ; Department of Biomedicine - Human Genetics, Aarhus University, Aarhus, Denmark |
| Broad/MHG | [aldumont@broadinsitute.org](mailto:aldumont@broadinsitute.org) | Dumont | Ashley |  |  | Stanley Center for Psychiatric Research, Broad Institute of Harvard and MIT, Cambridge, Massachusetts, USA |
| Broad/MHG | [jigold@broadinstitute.org](mailto:jigold@broadinstitute.org) | Goldstein | Jacqueline |  |  | Analytic and Translational Genetics Unit, Department of Medicine, Massachusetts General Hospital and Harvard Medical School, Boston, USA ; Stanley Center for Psychiatric Research, Broad Institute of Harvard and MIT, Cambridge, Massachusetts, USA ; Program in Medical and Population Genetics, Broad Institute of Harvard and MIT, Cambridge, Massachusetts, USA |
| iPSYCH | [grove@biomed.au.dk](mailto:grove@biomed.au.dk) | Grove | Jakob |  | PhD | The Lundbeck Foundation Initiative for Integrative Psychiatric Research, iPSYCH, Denmark ; Centre for Integrative Sequencing, iSEQ, Aarhus University, Aarhus, Denmark ; Department of Biomedicine - Human Genetics, Aarhus University, Aarhus, Denmark ; Bioinformatics Research Centre, Aarhus University, Aarhus, Denmark |
| iPSYCH | [CHSH@ssi.dk](mailto:CHSH@ssi.dk) | Hansen | Christine | S. |  | The Lundbeck Foundation Initiative for Integrative Psychiatric Research, iPSYCH, Denmark ; Center for Neonatal Screening, Department for Congenital Disorders, Statens Serum Institut, Copenhagen, Denmark ; Institute of Biological Psychiatry, MHC Sct. Hans, Mental Health Services Copenhagen, Denmark |
| iPSYCH | [DH@ssi.dk](mailto:DH@ssi.dk) | Hougaard | David | M. | DMSci | The Lundbeck Foundation Initiative for Integrative Psychiatric Research, iPSYCH, Denmark ; Center for Neonatal Screening, Department for Congenital Disorders, Statens Serum Institut, Copenhagen, Denmark |
| Broad/MHG | [howrigan@broadinstitute.org](mailto:howrigan@broadinstitute.org) | Howrigan | Daniel | P. | PhD | Analytic and Translational Genetics Unit, Department of Medicine, Massachusetts General Hospital and Harvard Medical School, Boston, Massachusetts, USA ; Stanley Center for Psychiatric Research, Broad Institute of Harvard and MIT, Cambridge, Massachusetts, USA |
| Broad/MHG | [hhuang@broadinstitute.org](mailto:hhuang@broadinstitute.org) | Huang | Hailiang |  | PhD | Analytic and Translational Genetics Unit, Department of Medicine, Massachusetts General Hospital and Harvard Medical School, Boston, Massachusetts, USA ; Stanley Center for Psychiatric Research, Broad Institute of Harvard and MIT, Cambridge, Massachusetts, USA |
| Broad/MHG | [julesm@gmail.com](mailto:julesm@gmail.com) | Maller | Julian |  | PhD | Stanley Center for Psychiatric Research, Broad Institute of Harvard and MIT, Cambridge, Massachusetts, USA ; Genomics plc, Oxford, UK |
| Broad/MHG | [armartin@broadinstitute.org](mailto:armartin@broadinstitute.org) | Martin | Alicia | R. | PhD | Analytic and Translational Genetics Unit, Department of Medicine, Massachusetts General Hospital and Harvard Medical School, Boston, USA ; Stanley Center for Psychiatric Research, Broad Institute of Harvard and MIT, Cambridge, Massachusetts, USA ; Program in Medical and Population Genetics, Broad Institute of Harvard and MIT, Cambridge, Massachusetts, USA |
| Broad/MHG | [jmartin@broadinstitute.org](mailto:jmartin@broadinstitute.org) | Martin | Joanna |  | PhD | Stanley Center for Psychiatric Research, Broad Institute of Harvard and MIT, Cambridge, Massachusetts, USA ; Department of Medical Epidemiology and Biostatistics, Karolinska Institutet, Stockholm, Sweden ; MRC Centre for Neuropsychiatric Genetics & Genomics, School of Medicine, Cardiff University, Cardiff, UK |
| iPSYCH | [mm@biomed.au.dk](mailto:mm@biomed.au.dk) | Mattheisen | Manuel |  | MD | The Lundbeck Foundation Initiative for Integrative Psychiatric Research, iPSYCH, Denmark ; Centre for Integrative Sequencing, iSEQ, Aarhus University, Aarhus, Denmark ; Department of Biomedicine - Human Genetics, Aarhus University, Aarhus, Denmark |
| Broad/MHG | [jennifermoran529@gmail.com](mailto:jennifermoran529@gmail.com) | Moran | Jennifer |  |  | Stanley Center for Psychiatric Research, Broad Institute of Harvard and MIT, Cambridge, Massachusetts, USA |
| iPSYCH | [nielmors@rm.dk](mailto:nielmors@rm.dk) | Mors | Ole |  | MD, PhD | The Lundbeck Foundation Initiative for Integrative Psychiatric Research, iPSYCH, Denmark ; Psychosis Research Unit, Aarhus University Hospital, Risskov, Denmark |
| iPSYCH | [pbm@econ.au.dk](mailto:pbm@econ.au.dk) | Mortensen | Preben | Bo | DMSci | The Lundbeck Foundation Initiative for Integrative Psychiatric Research, iPSYCH, Denmark ; Centre for Integrative Sequencing, iSEQ, Aarhus University, Aarhus, Denmark ; National Centre for Register-Based Research, Aarhus University, Aarhus, Denmark ; Centre for Integrated Register-based Research, Aarhus University, Aarhus, Denmark |
| Broad/MGH | [bneale@broadinstitute.org](mailto:bneale@broadinstitute.org) | Neale | Benjamin | M. | PhD | Analytic and Translational Genetics Unit, Department of Medicine, Massachusetts General Hospital and Harvard Medical School, Boston, USA ; Stanley Center for Psychiatric Research, Broad Institute of Harvard and MIT, Cambridge, Massachusetts, USA ; Program in Medical and Population Genetics, Broad Institute of Harvard and MIT, Cambridge, Massachusetts, USA |
| iPSYCH | [merete.nordentoft@dadlnet.dk](mailto:merete.nordentoft@dadlnet.dk) | Nordentoft | Merete |  |  | The Lundbeck Foundation Initiative for Integrative Psychiatric Research, iPSYCH, Denmark ; Mental Health Services in the Capital Region of Denmark, Mental Health Center Copenhagen, University of Copenhagen, Copenhagen, Denmark |
| iPSYCH | [jpal@biomed.au.dk](mailto:jpal@biomed.au.dk) | Pallsen | Jonatan |  | PhD | The Lundbeck Foundation Initiative for Integrative Psychiatric Research, iPSYCH, Denmark ; Centre for Integrative Sequencing, iSEQ, Aarhus University, Aarhus, Denmark ; Department of Biomedicine - Human Genetics, Aarhus University, Aarhus, Denmark |
| Broad/MHG | [dpalmer@broadinstitute.org](mailto:dpalmer@broadinstitute.org) | Palmer | Duncan | S. | PhD | Analytic and Translational Genetics Unit, Department of Medicine, Massachusetts General Hospital and Harvard Medical School, Boston, Massachusetts, USA ; Stanley Center for Psychiatric Research, Broad Institute of Harvard and MIT, Cambridge, Massachusetts, USA |
| iPSYCH | [cbp@econ.au.dk](mailto:cbp@econ.au.dk) | Pedersen | Carsten | Bøcker | DMSci | The Lundbeck Foundation Initiative for Integrative Psychiatric Research, iPSYCH, Denmark ; National Centre for Register-Based Research, Aarhus University, Aarhus, Denmark ; Centre for Integrated Register-based Research, Aarhus University, Aarhus, Denmark |
| iPSYCH | [mgp@econ.au.dk](mailto:mgp@econ.au.dk) | Pedersen | Marianne | Giørtz |  | The Lundbeck Foundation Initiative for Integrative Psychiatric Research, iPSYCH, Denmark ; National Centre for Register-Based Research, Aarhus University, Aarhus, Denmark ; Centre for Integrated Register-based Research, Aarhus University, Aarhus, Denmark |
| Broad/MHG | [tpoterba@broadinstitute.org](mailto:tpoterba@broadinstitute.org) | Poterba | Timothy |  |  | Analytic and Translational Genetics Unit, Department of Medicine, Massachusetts General Hospital and Harvard Medical School, Boston, USA ; Stanley Center for Psychiatric Research, Broad Institute of Harvard and MIT, Cambridge, Massachusetts, USA ; Program in Medical and Population Genetics, Broad Institute of Harvard and MIT, Cambridge, Massachusetts, USA |
| Broad/MHG | [sripke@broadinstitute.org](mailto:sripke@broadinstitute.org) | Ripke | Stephan |  | MD, PhD | Analytic and Translational Genetics Unit, Department of Medicine, Massachusetts General Hospital and Harvard Medical School, Boston, Massachusetts, USA ; Stanley Center for Psychiatric Research, Broad Institute of Harvard and MIT, Cambridge, Massachusetts, USA ; Program in Medical and Population Genetics, Broad Institute of Harvard and MIT, Cambridge, Massachusetts, USA ; Department of Psychiatry, Charite Universitatsmedizin Berlin Campus Benjamin Franklin, Berlin, Germany |
| Broad/MHG | [erob@broadinstitute.org](mailto:erob@broadinstitute.org) | Robinson | Elise | B. | PhD | Analytic and Translational Genetics Unit, Department of Medicine, Massachusetts General Hospital and Harvard Medical School, Boston, Massachusetts, USA ; Department of Epidemiology, Harvard Chan School of Public Health, Boston, Massachusetts, USA |
| Broad/MHG | [satterst@broadinstitute.org](mailto:satterst@broadinstitute.org) | Satterstrom | F. | Kyle |  | Analytic and Translational Genetics Unit, Department of Medicine, Massachusetts General Hospital and Harvard Medical School, Boston, USA ; Stanley Center for Psychiatric Research, Broad Institute of Harvard and MIT, Cambridge, Massachusetts, USA ; Program in Medical and Population Genetics, Broad Institute of Harvard and MIT, Cambridge, Massachusetts, USA |
| Broad/MHG | [stevens@broadinstitute.org](mailto:stevens@broadinstitute.org) | Stevens | Christine |  |  | Stanley Center for Psychiatric Research, Broad Institute of Harvard and MIT, Cambridge, Massachusetts, USA |
| Broad/MHG | [paturley@broadinstitute.org](mailto:paturley@broadinstitute.org) | Turley | Patrick |  | PhD | Analytic and Translational Genetics Unit, Department of Medicine, Massachusetts General Hospital and Harvard Medical School, Boston, Massachusetts, USA ; Stanley Center for Psychiatric Research, Broad Institute of Harvard and MIT, Cambridge, Massachusetts, USA |
| Broad/MHG | [rwalters@broadinstitute.org](mailto:rwalters@broadinstitute.org) | Walters | Raymond |  |  | Analytic and Translational Genetics Unit, Department of Medicine, Massachusetts General Hospital and Harvard Medical School, Boston, Massachusetts, USA ; Stanley Center for Psychiatric Research, Broad Institute of Harvard and MIT, Cambridge, Massachusetts, USA |
| iPSYCH | [Thomas.Werge@regionh.dk](mailto:Thomas.Werge@regionh.dk) | Werge | Thomas |  | PhD | The Lundbeck Foundation Initiative for Integrative Psychiatric Research, iPSYCH, Denmark ; Institute of Biological Psychiatry, MHC Sct. Hans, Mental Health Services Copenhagen, Denmark ; Department of Clinical Medicine, University of Copenhagen, Copenhagen, Denmark |
